# Degree of Phosphorus Saturation and Soil Phosphorus Thresholds in an Ultisol Amended with Triple Superphosphate and Phosphate Rocks

**DOI:** 10.1100/tsw.2011.131

**Published:** 2011-07-28

**Authors:** E. W. Gikonyo, A. R. Zaharah, M. M. Hanafi, A. R. Anuar

**Affiliations:** ^1^ Institute of Tropical Agriculture, Faculty of Agriculture, Universiti Putra Malaysia, Serdang, Selangor, Malaysia; ^2^ Kenya Agricultural Research Institute — Kabete, Nairobi, Kenya; ^3^ Department of Land Management, Faculty of Agriculture, Universiti Putra Malaysia, Serdang, Selangor, Malaysia

**Keywords:** phosphate rocks, triple superphosphate, degree of phosphorus saturation, phosphorus release, threshold point

## Abstract

Soil phosphorus (P) release capability could be assessed through the degree of P saturation (DPS). Our main objective was to determine DPS and, hence, P threshold DPS values of an Ultisol treated with triple superphosphate (TSP), Gafsa phosphate rocks (GPR), or Christmas Island phosphate rocks (CIPR), plus or minus manure. P release was determined by the iron oxide—impregnated paper strip (strip P), while DPS was determined from ammonium oxalate—extractable aluminum (Al), iron (Fe), and P. Soils were sampled from a closed incubation study involving soils treated with TSP, GPR, and CIPR at 0–400 mg P kg^-1^, and a field study where soils were fertilized with the same P sources at 100–300 kg P ha^-1^ plus or minus manure. The DPS was significantly influenced by P source x P rate, P source x manure (incubated soils), and by P source x P rate x time (field-sampled soils). Incubated soil results indicated that both initial P and total strip P were related to DPS by exponential functions: initial strip P = 1.38exp^0.18DPS^, R^2^ = 0.82** and total strip P = 8.01exp^0.13DPS^, R2 = 0.65**. Initial strip P was linearly related to total P; total P = 2.45, initial P + 8.41, R^2^ = 0.85**. The threshold DPS value established was about 22% (incubated soil). Field soils had lower DPS values <12% and strip P was related to initial DPS and average DPS in exponential functions: strip P = 2.6exp^0.44DPS^, R^2^ = 0.77** and strip P = 1.1DPS^2^ — 2.4DPS + 6.2, R^2^ = 0.58**, respectively. The threshold values were both at ≈8% and P release was 11–14 mg P kg^-1^. Results are evident that DPS can be used to predict P release, but the threshold values are environmentally sensitive; hence, recommendations should be based on field trials.

### INTRODUCTION

Phosphorus (P) is a major plant nutrient that limits agricultural production in many tropical and subtropical soils. On the other hand, it is an environmental risk factor responsible for P eutrophication in streams, lakes, and reservoirs because these systems are usually P-limited[1]. Nonetheless, P plays a pivotal role in freshwater environments, although it accounts for only 2–4% of the dry weight of most cells. Biomass production has been shown to be highly sensitive to small amounts of P[2] and for that reason, P losses from agricultural systems through surface runoff or subsurface drainage that are small in agronomic terms can have potentially serious implications for water quality[3]. Therefore, plant nutrition and environmental aspects must be taken into consideration in good fertilizer management.

One of the recent strategies proposed for sustainable intensification of food production in the tropics is “P recapitalization”[4]. This involves massive P application designed to permanently saturate the sorption sites and provide P for crop uptake. Therefore, the strategy turns the soil P-fixing capacity from a constraint into an asset. Fixed P forms the stock that provides P over a period of 5–10 years, referred to as soil capital[4]. Although “P recapitalization” looks attractive because of the economics involved due to escalating fertilizer costs over time, the consequences and limits of saturating the soils with P should be addressed. This is because accumulation of P partially or completely saturating the soil P sorption sites has been reported to increase leaching into subsurface soil layers[5] and increase P transported into surface waters via runoff[6]. Thus, excess levels of soil P have been linked with surface water quality degradation[7]. Consequently, the need for understanding the effects of different P sources and rates on P saturation and P release levels prior to “P recapitalization” is imperative.

Recent studies have shown that the degree of P saturation (DPS), a function of the portion of the soil exchangeable sites that are bound with P (P sorbed) in relation to the number of sites available for P binding capacity (PBC), is a good indicator of the soil’s potential to release P[8]. This concept was first introduced in the Netherlands, where it has been shown that, when a critical DPS level also referred to as threshold point is attained, there is a great probability of P release to the surface or groundwater soils. In the Netherlands, DPS-oxalate of 25% (i.e., groundwater P concentration >0.1 mg P $L^{-1}$ in soil solution) was established as a threshold for groundwater quality[9,10].

Phosphate fertilizers for agricultural production are generally classified as either water-soluble or water-insoluble P. The former includes triple superphosphate (TSP), single superphosphate (SSP), and diammonium phosphate (DAP), while the latter are mainly phosphate rocks (PRs). In Malaysia, PRs have been used almost exclusively as the P source for plantation crops for many years. Interest in PRs as direct-application fertilizer stems mainly from the fact that PRs are usually the cheapest fertilizer per kilogram of P. In addition, PRs are considered environmentally benign and sometimes used in many other countries where the organic farming practice is catching on. PRs are viewed as the P fertilizer most suitable for “P recapitalization” because of their cost compared to the expensive superphosphates (SPs), in addition to the fact that they are slow-release P fertilizers.

When P fertilizers are applied to the soil, phosphate ions are released into the soil solution followed by a fast and almost instantaneous fixation by the iron (Fe) and aluminum (Al) oxides in the soils. However, while the SPs dissolve immediately, releasing the P ions that are then fixed, thus influencing the DPS, the dissolution of different PRs varies widely due to the differences in physicochemical characteristics that influence their reactivity and their interactions with the soils and manure when applied in combination. The DPS of different PRs with or without manure will vary at different rates of PR application. However, most of the past studies on DPS have been conducted using SP- and organic manure–treated soils, and virtually no attempts have been made to evaluate the effects of PRs on DPS and P release as a result of different rates of application.

The objectives of the current study were therefore to: (1) determine the DPS at different rates of two PRs (Gafsa PR [GPR] and Christmas Island PR [CIPR]) compared to TSP, and (2) to establish the existence and behavior of the change points in soil P intensity (release into soil solution) for the differently treated soils.

## MATERIALS AND METHODS

### Phosphate Sources and Soil

Two experiments are reported in this study: one laboratory incubation and a field experiment. The major countries supplying PR to Malaysia are Egypt, Tunisia, Christmas Island, Australia, and Algeria, with values in proportion of 28.0, 22.5, 19.0, 16.0, and 14.5%, respectively[11]. The PRs selected for testing in this study were GPR, a carbonate-fluorapatite deposit from Tunisia, representing the reactive PR, and Christmas Island PR (CIPR) A-grade, PR (A-ore) dominated by hydroxyl-fluor-carbonate apatite as the nonreactive PR.

Soil used for the incubation experiment was sampled from the same site where the field experiment was conducted. The site was Puchong farm, of the Universiti Putra Malaysia (UPM) in Serdang, Selangor ($2^o$ 59' N and $101^o$ 38' E, 35 m above sea level). The soil was classified as Bungor soil series, an Ultisol (Typic Kandiudult, clayey, kaolinitic, isohyperthermic). Puchong (experimental site) is characterized by an average annual precipitation of about 2,200 mm, and mean monthly maximum and minimum temperatures of 32 and 24°C, respectively, and a relative humidity of 70–90%. Characterization of the soil, fertilizers, and manure were done by standard methods[12] prior to the beginning of the experiments. Briefly, (1) total carbon was determined using the total carbon analyzer (TOC) model Leco CR-412; (2) available P was determined by Bray 1 (0.03 M $NH_4F$ + 0.025M HCl), Fe oxide strip P (1:40 ratio of soil to 0.02 M KCl + Fe-oxide–coated filter paper strip; 16-h reaction time, followed by desorption of P with 1 M $H_2SO_4$), and Olsen P (0.5 N $NaHCO_3$ at pH 8.5); (3) total soil P was determined by digestion with 18% $HClO_4$; (4) P in all extracts was determined by the molybdenum blue method using ascorbic acid as the reducing agent; (5) cation exchange capacity (CEC) was determined by leaching with 1 M $NH_4OAc$ at pH 7.0 followed by 0.5 M $K_2SO_4$; (6) acid ammonium oxalate–extractable $Al_2O_3$ and $Fe_2O_3P$ were measured by atomic absorption spectrophotometry (AAS); and (7) fertilizers and manure elemental contents were determined by digestion with 18% perchloric acid.

### Closed Incubation Experiment

Soil samples (100 g, air dried, sieved through 2 mm) in triplicates were treated with TSP, GPR, and CIPR at four rates each: 0, 100, 200, and 400 mg P $kg^{-1}$, which are equivalent to about 200–800 kg P $ha^{-1}$ in the field. Soils were wetted to approximate field capacity for 4 days at room temperature (28 ± 3°C), and then air dried for another 3 days. The wetting and drying cycles were repeated 13 times (13 × 7), thus 91 days. The wetting and drying cycles were to simulate field environmental conditions. After the first cycle, soils were mixed to ensure uniform distribution of P. At the end of the equilibration period, soils were air dried and sieved (2 mm), and P release was determined by sequential P extraction using the Fe-impregnated paper strip (strip P). The soils were also analyzed for ammonium oxalate–extractable P, Al, and Fe for DPS determination.

### P Extraction by the Strip P Method

Sequential extraction of P using Fe oxide–impregnated paper strips (strip P method) was made possible by the "nondisruptive" nature of the paper strip. The consecutive extraction of soil P by the strip P is considered a convenient laboratory method to characterize the capacity of a soil to supply P or to investigate the kinetics of P release[13]. P extraction was repeated nine times consecutively. The strip P were prepared according to the procedure described by Menon et al.[14]. They were prepared by passing filter paper No. 541 swiftly through a 10% $FeCl_3$ solution. The papers were air dried and then treated with ammonium vapor to oxidize the Fe chloride on the paper to Fe oxide. The papers were then air dried, cut into 10- × 2-cm strips, and stored for subsequent use.

P was extracted from the soil by shaking a 1-g soil sample and one paper strip in 40 mL of 0.02 M KCl end-over-end for 16 h. The strip was removed from the solution, rinsed free of adhering soil particles, and air dried. P retained on the strip was eluted from the strip by shaking it end-over-end with 40 mL of 0.1 M $H_2SO_4$ for 2 h[15]. The process was repeated eight times by adding a fresh paper strip each time to the same soil solution and then eluting the P from the strip as described above.

### Determination of the DPS

Soils were extracted by acid ammonium oxalate because this extractant is designated to remove the P associated with amorphous oxides of Al and Fe (the two principal components) of P sorption in noncalcareous acid soils. Total Al, Fe, and P in the acid ammonium oxalate[16] were determined by inductively coupled plasma spectrometry, and the DPS calculated as 100 × (P/0.5(Al + Fe), where P, Fe, and Al are in mmol $kg^{-1}$, and DPS is expressed as a percentage[17]. The sum of ammonium oxalate Al and Fe, designated as Ox-Al and Ox-Fe, respectively, provides an indicator of the P sorption capacity (PSC) of the acid soil.

The procedure involved extracting P, Fe, and Al in 0.2 M ammonium oxalate at pH 3.0 at a soil:solution ratio of 1:50 with 4 h of shaking in the dark. This was followed by the addition of "superfloc 127", a flocculating compound to facilitate infiltration[18]. The supernatants collected were then diluted by 1:20 before reading them on the inductively coupled argon spectrophotometer (ICAP)(Model: Thermal Elemental IRIS Advantage) for total Fe, Al, and P. The DPS was calculated for each soil sample and P release determined through the sequential strip P method. The initial P and total P released were then regressed against the DPS of the soil to establish the relationship between the two.

### Field Experiment

Setaria grass (*Setaria anceps Stapf. cv. "Kazungula*") was used as the test crop in this experiment because it has widespread occurrence and can survive in low soil fertility, but also responds well to N and P application. Additionally, it is persistent under frequent cutting or grazing; hence, it was suitable for the sequential harvesting required in the experiment. Experimental treatments for assessment of Setaria grass dry matter response to P from different sources and rates were applied as factorial combinations consisting of 20 treatments ([nil + (3 P fertilizer sources × 3 P rates)] × 2 levels of manure) with three replications arranged in a randomized complete block design (RCBD). Similar P sources to those used in the incubation experiment were used. Fertilizers were applied at the rates 100, 200, and 300 kg P $ha^{-1}$ alone (sole) or combined with cattle manure at zero (0) or 20 t $ha^{-1}$. Fertilizer rates were based on previous rates tested in "P recapitalization" studies in tropical acid soils[19].

The experimental plots measured 1.5 × 1.5 $m^2$, and the P fertilizers and manure were applied once at the beginning of the experiment by incorporating them into the top 10-cm depth of the soil 2 weeks prior to planting. At planting and after every harvest, all plots received a basal application of N and K at 60 kg N $ha^{-1}$ as urea and 100 kg K $ha^{-1}$ as muriate of potash (KCl), respectively. Setaria grass was established from clean seeds obtained from Malaysia Agricultural Research and Development Institute (MARDI). Harvesting and soil sampling were done at 2-month intervals for a period of 8 months. Soil sampling was done by taking composite soil samples at 0–15 cm before the experiment commenced and after each harvest. Subsamples were taken out of a thorough mixture of six to eight soil cores per plot, taken in a criss-cross fashion using a 6-cm-diameter soil auger. The soils were air dried in the laboratory and then ground with a mortar and pestle to pass through a 2-mm sieve in preparation for analysis.

### Statistical Analysis

All data were analyzed using Statistical Analysis System, version 8.1[20]. Analysis of variance (ANOVA) was performed using the PROC MIXED procedure[21]. Field experiment data involving repeated soil sampling (time 0 to time 4) were subjected to combined analysis of repeated measures using the mixed model procedure. Selection of an appropriate covariance structure of the data was done by trying various structures and examining the goodness-of-fit measures for the different structures in order to obtain the correct standard errors. The covariance structures with the smallest Schwarz's Bayesian Criterion and Akaike's Information Criterion were selected, and the appropriate analysis was conducted using the selected structure. Regression between P released (strip P) and DPS was done by plotting curves using Excel and selecting the best-fitted model on the basis of the highest $R^2$. The change points in the curve slopes (threshold points) were determined graphically from a plot of strip P against DPS. They were determined by a plot of the tangent on the two parts of the curve (the lower part and the upper part with different slopes), and the point where the two tangents intersected was considered the threshold DPS point and the corresponding P concentration as the critical P release level. Comparison between treatment means was done using Duncan's multiple range test (DMRT).

## RESULTS

### Soils, Fertilizers, and Manure Characterization

Characterization results for soils, fertilizers, and manure are presented in Tables 1A and 1B, respectively. In addition to the manure results presented in Table 1B, manure also exhibited the following characteristics: dissolved organic carbon (DOC) 21 g $kg^{-1}$, C/N ratio 27; humic acid content 10.2 g $kg^{-1}$, and fulvic acid content, traces.

**TABLE 1A**
**Characteristics of the Top- and Subsoil**

| Parameter | Soil Characteristic |
|---|---|
| pH water (1:2.5) | 4.5 |
| pH KCl (1:2.5) | 3.9 |
| Total C (g $kg^{-1}$) | 30 |
| Total P (mg P $kg^{-1}$ soil) | 280 |
| P sorption maximum (mg P $kg^{-1}$ soil) | 769 |
| Available P (mg P $kg^{-1}$ soil) by: | |
| Bray 1 | 4.9 |
| Olsen | 5.2 |
| Strip P | 0.6 |
| $Al_2O_3$ (mg $kg^{-1}$) | 1520 |
| $Fe_2O_3$ (mg $kg^{-1}$) | 3080 |
| CEC (cmol [+] $kg^{-1}$) | 5.4 |

**TABLE 1B**
**Characteristics of the Fertilizers Used**

| Composition | Fertilizers and Manure (g kg$^{-1}$) | | | |
|---|---|---|---|---|
| | TSP | GPR | CIPR | Manure |
| P | 204 | 125 | 140 | 10 |
| CaO | 192 | 455 | 420 | 50 |
| $Fe_2O_3$ | 6 | 4 | 31 | 3 |
| $Al_2O_3$ | 16 | 9 | 107 | 0.04 |
| MgO | 29 | 9 | 23 | 11 |
| Solubility in water* | 380 | 0.2 | 0.1 | N/D† |
| Solubility in 2% citric acid* | 430 | 100 | 87 | N/D† |
| Solubility in 2% formic acid* | 407 | 110 | 66 | N/D† |

* Solubility based on total $P_2O_5$.

† Not determined.

### Incubation Studies

#### *Effect of Treatments on Ammonium Oxalate–Extractable Al, Fe, P, and DPS*

Statistical analysis of the incubated soils indicated that addition of the different P sources resulted in significant differences in Ox-Fe and Ox-Al ($p = 0.01$). The TSP-treated soils gave the highest Ox-Al and Ox-Fe (2776 and 3918 mg kg$^{-1}$, respectively), and the amounts were not significantly different from those extracted from CIPR-treated soils (Table 2A). The least were extracted from GPR treatments (2270 and 3272 mg kg$^{-1}$, respectively, for Ox-Al and Ox-Fe (Table 2A). Thus, Ox-Fe was more than Ox-Al. Unlike Ox-Al, Ox-Fe was also significantly influenced by the P rate ($p = 0.023$) and manure ($p = 0.01$) (Table 2A).

The sum of the two oxides (Al+Fe-Ox), an important estimate of the PSC of the soil, was significantly influenced by the P source ($p < 0.01$), manure addition ($p = 0.07$), and the interaction of P source × P rate × manure ($p = 0.15$). On average, TSP treatments gave the highest amounts of Al+Fe-Ox (6693 mg kg$^{-1}$) and were not significantly different from CIPR treatments, while GPR treatments gave the least amounts (5542 mg kg$^{-1}$). The sum of the two oxides also varied with the fertilizer rates, but did not show a clear trend. For instance, based on fertilizer rates, the highest amount of Al+Fe-Ox (6585 mg kg$^{-1}$) was attained at the highest fertilizer rate (400 mg P kg$^{-1}$), while the lowest (5814 mg kg$^{-1}$) was attained at 100 mg P kg$^{-1}$. Manure addition increased Al+Fe-Ox by about 12%. However, ultimately, the amounts of Al+Fe-Ox were determined by the interaction of P source × P rate × manure ($p = 0.01$), as shown in Fig. 1a.

From the plots, it was observed that Al+Fe-Ox extracted from sole manure treatments were very high, ≈45% more than the amounts extracted from the control (nil treatment). An integration of the different P sources with manure led to amounts of Al+Fe-Ox that varied with the P sources and fertilizer rates. For example, at the 100 mg P kg$^{-1}$ fertilizer rate, the sole P sources exhibited lower amounts of Al+Fe-Ox (≈5200–5500 mg kg$^{-1}$) than those integrated with manure (≈6000 mg kg$^{-1}$), with the exception of GPR (Fig. 1a).

At the 200 mg P kg$^{-1}$ rate, the sole TSP and CIPR treatments showed higher amounts of Al+Fe-Ox (6500–7300 mg kg$^{-1}$) than their manure combinations (≈6000 mg kg$^{-1}$), while the sole GPR and manure/GPR combination both showed relatively lower amounts, ≈5000 mg kg$^{-1}$ (Fig. 1a). At the 400 mg P kg$^{-1}$ rate, Al+Fe-Ox levels from CIPR and TSP treatments increased almost linearly to ≈7500–8000 mg kg$^{-1}$, while in the sole P sources, amounts remained constant (Fig. 1a). Conversely, in both the sole GPR and GPR/manure combination, Al+Fe-Ox levels remained the same (Fig. 1a).

**TABLE 2A**
**Effect of Treatments on Ammonium Oxalate (pH = 3.0)–Extractable Al, Fe, P, and DPS from the Incubated Soils**

| Treatments | Ammoniun Oxalate Extractable Elements | | | | |
|---|---|---|---|---|---|
| | Al | Fe | Al + Fe | P | DPS |
| | (mg kg$^{-1}$) | | | | (%) |
| P sources | | | | | |
| CIPR | 2634a | 3778a | 6411a | 225b | 10.7c |
| GPR | 2270b | 3272b | 5542b | 245b | 18.3a |
| TSP | 2776a | 3918a | 6693a | 343a | 13.9b |
| Fertilizer rates | | | | | |
| Control | 2673a | 3692ab | 6365ab | 121d | 5.4d |
| 100 (mg P kg$^{-1}$) | 2445a | 3369b | 5814b | 207c | 11.8c |
| 200 (mg P kg$^{-1}$) | 2438a | 3660ab | 6097ab | 298b | 17.9b |
| 400 (mg P kg$^{-1}$) | 2684a | 3903a | 6585a | 457a | 22.1a |
| Manure | | | | | |
| Without manure | 2480a | 3374b | 5853b | 230b | 12.9b |
| With manure | 2640a | 3938a | 6577a | 312a | 15.7$^{a}$ |

*Note*: Numbers with the same letters in the same column under the same treatment are not significantly different. Means separated by DMRT at 5% level.

Oxalate P (Ox-P) was significantly influenced by the P sources ($p < 0.01$), P rates ($p < 0.01$), manure addition ($p < 0.01$), and their interactions ($p < 0.01$–1.46). The highest average Ox-P (343 mg P kg$^{-1}$) was extracted from TSP treatments, while the least (≈225 mg kg$^{-1}$) was extracted from CIPR, but the latter was not significantly different from GPR treatments (Table 2A). The Ox-P also increased in accordance with the increasing rates of fertilizer application. Increasing the rate of fertilizer application from 0 (control) to 400 mg P kg$^{-1}$ led to a 214% increase in Ox-P relative to control. The addition of manure increased Ox-P by about 36%. However, the interactions of P source × P rate × manure determined the ultimate Ox-P, and a plot of the different P rates with and without manure addition against fertilizer P rate were drawn to show the trends of the various treatments.

The plot revealed that Ox-P extracted from sole TSP and TSP/manure combination exhibited linear relationships with fertilizer rates, whereby the latter showed higher Ox-P levels than sole TSP (Fig. 1b). On the other hand, Ox-P extracted from the PRs exhibited quadratic relationships with fertilizer rate and attained the maxima at 200 mg P kg$^{-1}$ and remained constant up to 400 mg P kg$^{-1}$ (Fig. 1b). However, it was noted that a combination of manure and GPR gave remarkably higher Ox-P than sole GPR, but effects declined to almost 0 or insignificant levels at 400 mg P kg$^{-1}$ (Fig. 1b). The CIPR/manure combination showed the highest Ox-P at the 100 mg P kg$^{-1}$ rate and declined with increasing fertilizer rate (Fig. 1b).

The DPS was significantly influenced by the P sources ($p < 0.01$), P rate ($p < 0.01$), manure ($p = 0.02$), and the interaction, P source × P rate ($p < 0.01$). On average, DPS from the three P sources ranged from 10.7% (CIPR) to 18.3% (GPR). The DPS increased with increasing P rates from an average of 5.4% (control) to 22% (400 mg P kg$^{-1}$). Manure application led to increased DPS from an average of 12.9% (minus manure treatments) to 22.1% (plus manure treatments). However, a plot of %DPS resulting from different treatment combinations against fertilizer P rates was drawn to examine the effects of specific treatment combinations and how they changed with varying P rates. The plots exhibited quadratic

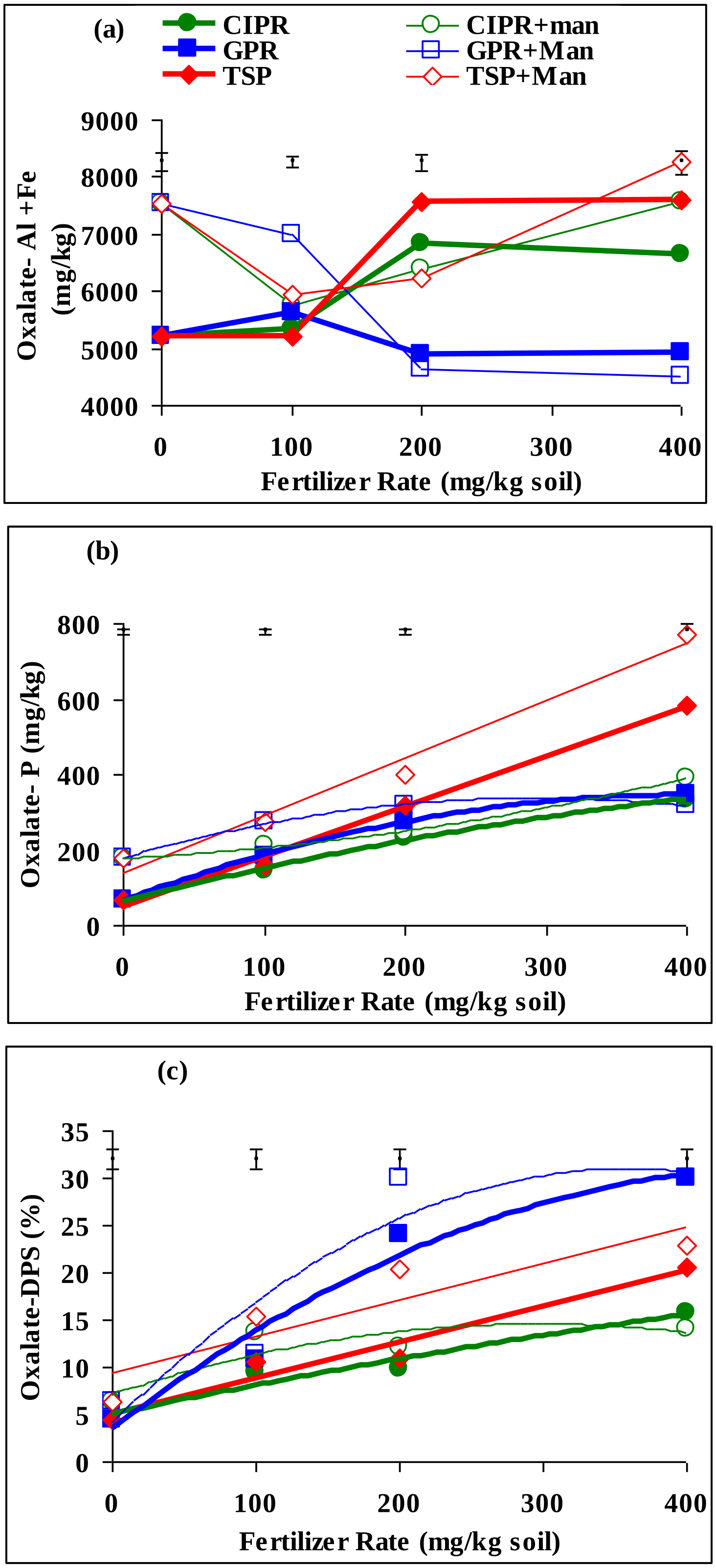

**FIGURE 1.** Effects of P source × P rate × manure on (a) oxalate Al + Fe, (b) oxalate P, and (c) DPS.

relationships for the sole PRs and PR/manure combinations, and linear relationships for sole TSP and TSP/manure combinations (Fig. 1c). The highest DPS was shown by GPR and GPR + manure, while CIPR had the lowest (Fig. 1c). It was noted that the CIPR/manure combinations at 100–200 mg P $kg^{-1}$ gave higher DPS values than sole TSP (Fig. 1c).

### Sequentially Extracted P Using the Strip P

Individual analysis of the nine strip P extractions indicated that the effects of P source and P rate were significant through all, while the effect of manure was only significant in the first and last two extractions. In addition, the effects of interactions P sources × P rate ($p < 0.01$), P sources × manure ($p = 0.034$), and P sources × P rate × manure ($p < 0.01$) were all significant.

Results of combined analysis over time indicated that the P sources, P rate, manure addition, and their interaction effects were significant. On average, GPR yielded the highest strip P (3.93 mg P $kg^{-1}$), although not significantly different from TSP, while CIPR yielded the lowest (51% of GPR). The strip P increased with the P rates from 1.2 mg P $kg^{-1}$ (control) to about 400% relative to control (at the 400 mg P $kg^{-1}$ rate). On average, manure increased strip P by about 19% of the control.

However, the interaction of P source × P rate × manure × strip number determined the ultimate strip P. Plots of strip P extracted against the strip number for the different P sources at different rates with and without manure revealed that the plots were described generally by exponential functions described by the equations:

$$\text{Strip P} = a{*}e^{-bx} \text{ for PRs}$$

$$\text{Strip P} = ax^{-b} \text{ for TSP (Fig. 2a–c)}$$

where the constants “a” and “b” were related to initial strip P and rate of P release, respectively, and “x” represented the extraction number. Sole manure treatment was not significantly different from control (not shown). At 100 mg P $kg^{-1}$, there were no significant differences between the sole P sources and their combination with manure except at extraction one; hence, only plots of P sources integrated with manure are shown in Fig. 2a. At 200 mg P $kg^{-1}$, GPR yielded the highest P among the three P sources with an exception of the first extraction, in which case TSP yielded the highest (Fig. 4b). However, when integrated with manure, GPR yielded the highest P from extraction one through all to the ninth (Fig. 4b). Manure addition increased strip P in the two PRs in all the extractions, but in TSP, manure increased strip P only in the first two extractions. The most striking observation was the remarkably high increase in strip P from the CIPR/manure combination to comparable amounts with TSP at 200 mg P $kg^{-1}$ (Fig. 4b). At the highest P rate (400 mg P $kg^{-1}$), the GPR/manure combination continued to yield the highest strip P through all, but contrarily, the effects of manure combination with TSP or CIPR were almost negligible at this rate (Fig. 2c).

### Effects of DPS on Initial Strip P (Strip Number One Extractable P) and the Total Strip P Released

Initial P or P extracted by the first strip was related to DPS by an exponential relationship described by the equation:

$$\text{Initial strip P} = 1.38\exp^{0.18\text{DPS}}, R^2 = 0.82^{**}$$

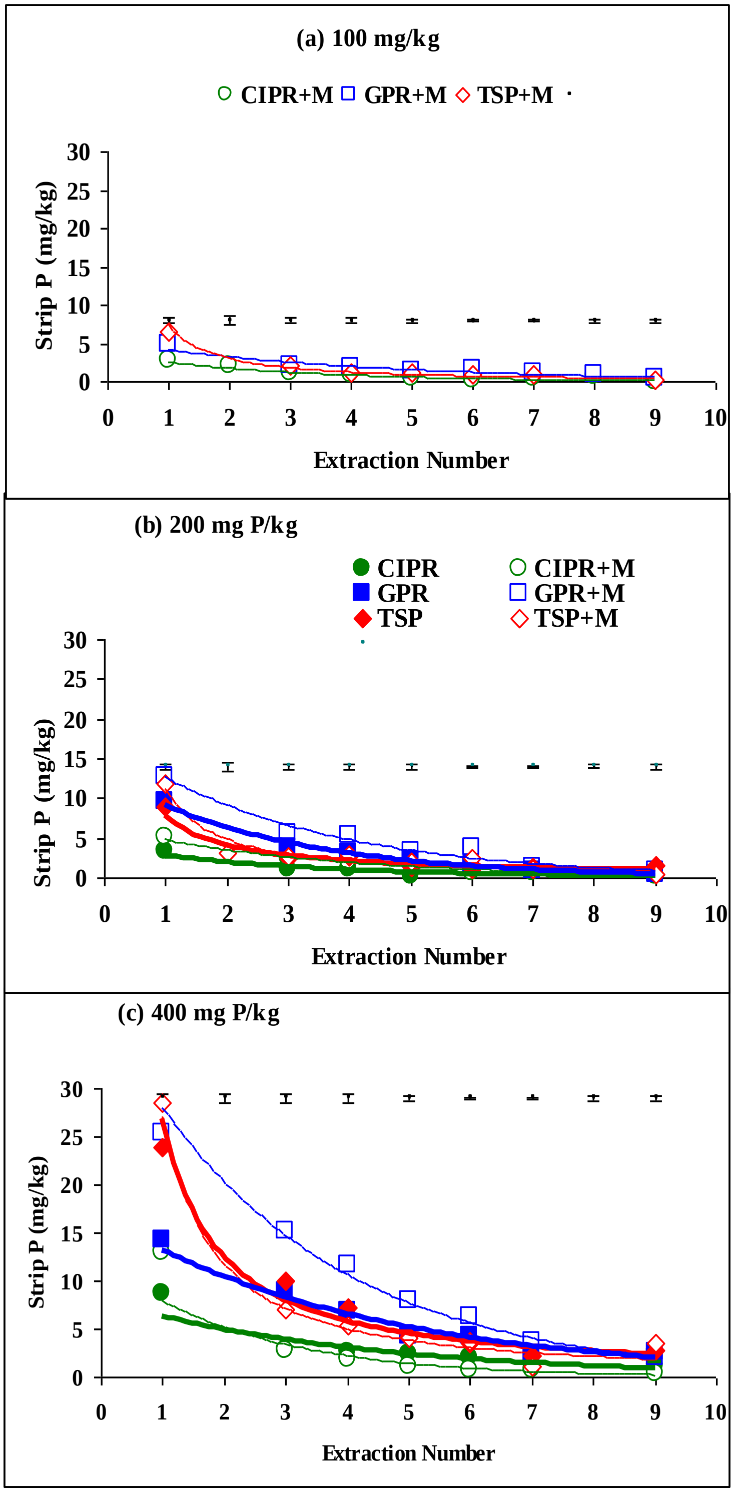

**FIGURE 2**. Effects of P source × manure × strip number on strip P at fertilizer rate (a) 100 mg P $kg^{-1}$, (b) 200 mg P $kg^{-1}$, and (c) 400 mg P $kg^{-1}$. Note: sole P sources were not presented in (a).

The rate of P release at the lower end of the curve was quite low (≈0.4 mg P $kg^{-1}$ for 1% change in DPS) below 22% DPS and increased by about fivefold (2.1 mg $kg^{-1}$ for 1% change in DPS) above that point (Fig. 3a). The point of slope change 22% DPS corresponded to P release from the first strip of 12 mg P $kg^{-1}$. Similarly, total strip P released (by nine strips) was related to the DPS and likewise the two were related by an exponential relationship described by the equation:

$$\text{Total strip P} = 8.01\exp^{0.13\text{DPS}},\ R^2 = 0.66^{**} \text{ (Fig. 3b)}$$

The plot also exhibited a change of slope at almost the same DPS value (21% DPS). The change in P release was more than threefold from 1.1 to 3.6 mg $kg^{-1}$ for 1% change in DPS (Fig. 3b). The P intensity was much higher (about 40 mg P $kg^{-1}$) for the total P (Fig. 3b). The initial strip P and the total were linearly and strongly related as described by the equation:

$$\text{Total strip P} = 2.45 \text{ Initial strip P} + 8.41,\ R^2 = 0.85^{**} \text{ (Fig. 3c)}$$

### Field Experiment

#### *Oxalate-Extractable Al, Fe, P, and DPS*

Lower average amounts of Ox-Al (1739–2106 mg Al $kg^{-1}$) than Ox-Fe (2446–2736 mg Fe $kg^{-1}$ ) were extracted from the treated soils, with the highest and lowest amounts extracted from control and highest fertilizer rate, respectively (Table 2B). Amounts of Ox-Al extracted were influenced significantly by the rate of fertilizer application and varied significantly over time, but the average amounts were not influenced by the fertilizer type or the addition of manure (Table 2B). On the other hand, amounts of Ox-Fe extracted were not significantly influenced by any of the treatments except time ($p < 0.01$), but the P rate × time interaction was significant ($p = 0.1$). However, to examine the specific treatment effects on the sum of Al+Fe-Ox, a plot of P source × P rate × time was constructed.

From the plots of P source × P rate × time for Al+Fe-Ox, Ox-P, and Ox-DPS (Fig. 4a–c), it was observed that Al+Fe-Ox declined with time up to the sixth month and afterwards seemed to increase. Some obvious differences between the different P sources in relation to amounts of Al+Fe-Ox extracted were observed. At the lower rate of fertilizer application, the lowest amount of Al+Fe-Ox was extracted from TSP (2000–2280 mg $kg^{-1}$) and the highest from GPR (1970–3430 mg $kg^{-1}$) for the first 6 months (Fig. 4a). However, after the sixth month, the amount of Al+Fe-Ox extracted from all the sources increased with the highest amounts from TSP ≈ CIPR, while lower amounts were extracted from GPR (Fig. 4a).

At the highest fertilizer P application rate, the highest amount of Al+Fe-Ox was extracted from TSP (3580–2340 mg $kg^{-1}$) as shown in Fig. 4b. The PRs exhibited lower and then later higher amounts of Al+Fe-Ox than TSP, but the two PRs were not significantly different (Fig. 4b).

Extractable Ox-P was significantly influenced by fertilizer source ($p = 0.27$) and fertilizer rate ($p < 0.01$), but not influenced by manure addition. The only significant interactions on Ox-P were fertilizer source × fertilizer rate, and P fertilizer rate × time. On average, CIPR-treated soil resulted in the lowest extracted amount of Ox-P (80.1 mg P $kg^{-1}$), while TSP resulted in the highest (111 mg P $kg^{-1}$). Average extracted Ox-P from the control was 61.8 mg P $kg^{-1}$ and increased twofold, when P rate was increased to 300 kg P $ha^{-1}$ (Table 2B).

The plots of Ox-P extracted from the different P sources × fertilizer rate × time indicated that at the 100 kg P $ha^{-1}$ rate, amounts of Ox-P extracted from the three P sources did not show significant differences, although for the first 2 months, slightly higher amounts of P were extracted from GPR than TSP/CIPR. Nevertheless, after the fourth month, amounts extracted from TSP were slightly higher than from other sources (Fig. 4c). At the highest P application rate, TSP resulted in the highest amounts of Ox-P, significantly different from the two PRs for the entire experimental period (Fig. 4d). The two PR treatments

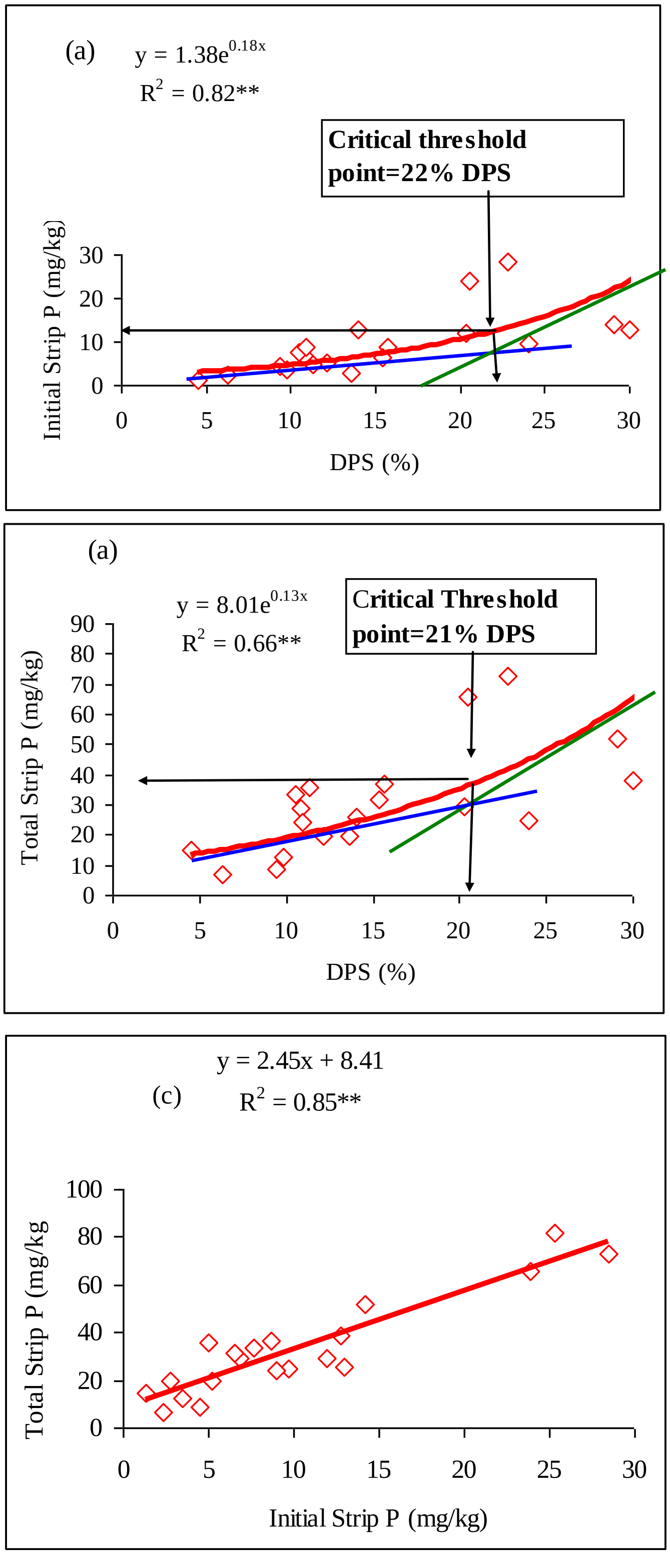

**FIGURE 3.** Relationship between (a) initial strip P vs. DPS, (b) total strip P (nine strips) vs. DPS, (c) total strip P vs. initial strip P. Note: different scales for a, b, and c to enable easy plotting of the threshold points. The blue and green lines in a and b are the tangents for the lower and upper slopes, respectively. The point where the two intersect is considered the change point of the slope and the corresponding strip P is the critical P intensity.

gave Ox-P levels that were not statistically different, although they gradually increased with time through all the experimental period from about 70 mg P $kg^{-1}$ to 130–160 mg P $kg^{-1}$(Fig. 4d).

**TABLE 2B**
**Effect of Treatments on Ammonium Oxalate (pH=3.0)–Extractable Al, Fe, and P from the Field-Sampled Soils**

| | Al ± S.E | Fe ± S.E | (Al+Fe) ± S.E | P ± S.E |
|---|---|---|---|---|
| | (mg kg$^{-1}$) | | | |
| Fertilizer type | | | | |
| CIPR | 1896 ± 78.5 | 2601 ± 133.2 | 4497 ± 195.4 | 80 ± 6.1 |
| GPR | 1892 ± 78.5 | 2659 ± 133.2 | 4551 ± 195.4 | 80 ± 6.1 |
| TSP | 1996 ± 78.5 | 2626 ± 133.2 | 4622 ± 195.4 | 111 ± 6.1 |
| Fertilizer rates | | | | |
| 0 (control) | 1739 ± 78.5 | 2443 ± 133.2 | 4182 ± 195.4 | 62 ± 6.1 |
| 100 (all fertilizers) | 1939 ± 78.5 | 2706 ± 133.2 | 4445 ± 195.4 | 90 ± 6.1 |
| 300 (all fertilizers) | 2106 ± 78.5 | 2736 ± 133.2 | 4842 ± 195.4 | 128 ± 6.1 |
| Manure addition | | | | |
| All fertilizers without manure | 2002 ± 64.1 | 2658 ± 133.2 | 4660 ± 159.6 | 87 ± 5.0 |
| All fertilizers with manure | 1854 ± 64.1 | 2599 ± 133.2 | 4453 ± 159.6 | 99 ± 5.0 |

### *DPS*

DPS were significantly influenced by P fertilizer source ($p < 0.04$), P rate ($p < 0.01$), manure addition ($p < 0.01$), and the fertilizer source × P rate × time interaction ($p < 0.01$). The DPS values were generally low (due to the extracted high levels of Fe and Al relative to P). On average, TSP gave the highest average DPS level (5.2%), while CIPR treatments resulted in the lowest (4.3%). The DPS also increased with fertilizer rate from 3.6% (control) to 6.0% (300 kg P ha$^{-1}$). Manure addition also increased DPS from an average of 4.3 (minus manure) to 5.0% (plus manure).

Plots of different P sources × fertilizer rate × time revealed that at the 100 kg P ha$^{-1}$ rate of fertilizer application, TSP and GPR treatments gave DPS levels that were not significantly different in the entire experimental duration (Fig. 4e). In addition, it was observed that for the three fertilizers, DPS increased with time for the first 4 months (>4%) and then declined gradually to the eighth month (Fig. 4e). At the highest P rate, TSP showed the highest DPS significantly different from the PRs, while CIPR and GPR gave lower DPS levels that were not significantly different (Fig. 4f).

### *Fe Oxide–Impregnated Paper Strip P*

Extractable P using strip P was not sensitive to fertilizer type and manure addition, but was significantly influenced by P fertilizer rate ($p < 0.01$). The magnitude of strip P was small, ranging from an average of 7.3 mg P kg$^{-1}$ (GPR) to 8.4 mg P kg$^{-1}$(TSP) in all the fertilizer treatments. Strip P increased with fertilizer rate from an average of 4.3 (control) to 12.6 mg P kg$^{-1}$ (300 kg P ha$^{-1}$). Over time, an interaction of P source × fertilizer rate × manure influenced the ultimate strip P levels significantly. From the plot of the interaction, it was observed that strip P declined relatively fast from ≈13 mg P ha$^{-1}$ (second month) to ≈5 mg P ha$^{-1}$ (sixth month) at the lower P rate (Fig. 5a). Strip P from sole TSP treatments seemed the highest among all the sources (Fig. 5b). Similarly, at the highest fertilizer rate, the strip P levels also declined fast from the second month to the sixth month and remained relatively constant thereafter (Fig. 5b). The three P sources/manure combinations gave higher amounts of strip P than the corresponding sole P sources (Fig. 5b). When the PRs were combined with manure, they yielded comparable amounts of strip P to TSP (Fig. 5b).

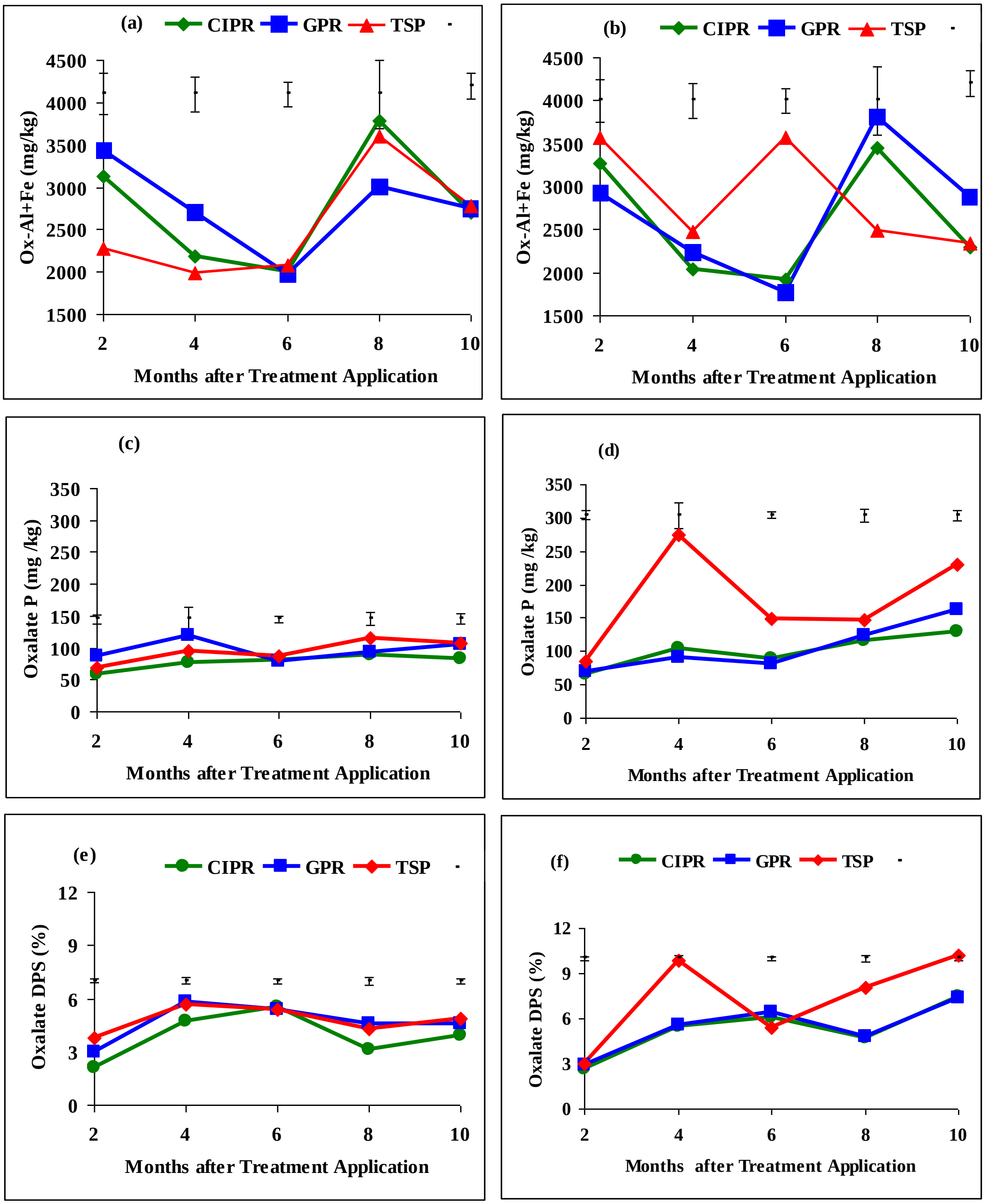

**FIGURE 4.** Effects of P source × time at 100 and 300 kg P ha$^{-1}$, respectively, on (a,b) oxalate Al + Fe, (c,d) oxalate P, and (e,f) oxalate DPS.

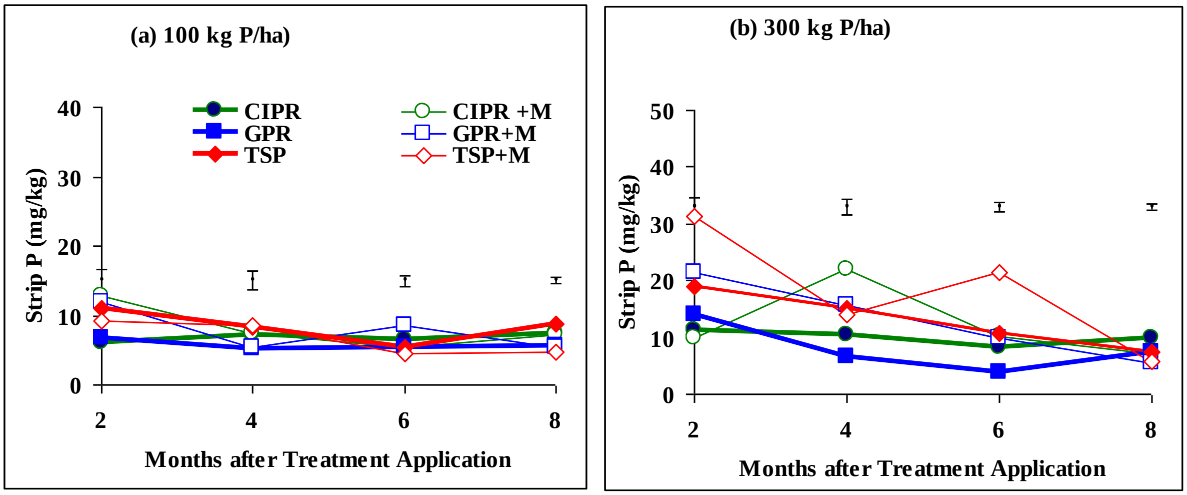

**FIGURE 5.** Effects of P source × manure × time on strip P release at (a) 100 kg P $ha^{-1}$ and (b) 300 kg P $ha^{-1}$.

#### Relationship between DPS and the Strip P

The initial DPS, 2 months after application, was related to the strip P by an exponential relationship:

$$\text{Strip P} = 2.6\exp^{0.44\text{DPS}}, R^2 = 0.77^{**}$$

The rate of P release at the lower end of the curve was low (1.4 mg P $kg^{-1}$ for 1% change in DPS), below 8% DPS, and increased by about threefold (4.2 mg $kg^{-1}$ for 1% change in DPS) above that point (Fig. 6a). The point of change corresponded to a P release of ≈14 mg P $kg^{-1}$. The average strip P was also related to average DPS by an exponential-type equation:

$$\text{Strip P} = 1.1\text{DPS}^2 - 2.4\text{DPS} + 6.2, R^2 = 0.54^{**}$$

There was a distinct change of slope greater than fourfold (0.6–2.7 mg P $kg^{-1}$ for 1% change in DPS) from the lower slope to the upper slope of the curve (Fig. 6b). The change point was about 8% DPS corresponding to P release of about 11 mg P $kg^{-1}$.

### DISCUSSION

#### Oxalate-Extractable Fe, Al, P, and DPS in the Closed Incubation Experiment Soils

Oxalate is known to extract mainly the noncrystalline or amorphous Fe and Al[22]. Results from the incubation study revealed that Ox-Al and Ox-Fe were influenced by P sources, probably associated in part to the Al and Fe contents of the P sources and partly that from the soil. For example, TSP- and CIPR-treated soils resulted in the highest Ox-Al and Ox-Fe, which were not significantly different and could probably be attributed to the higher $Al_2O_5$ and $Fe_2O_5$ contents in TSP (16 and 6 g $kg^{-1}$, respectively) and CIPR (31 and 107 mg $kg^{-1}$, respectively) compared to GPR (4 and 9 g $kg^{-1}$, respectively) as shown in Table 1B, also reported in Gikonyo et al.[23]. Although TSP had much lower Al and Fe contents than CIPR, its solubility was much higher compared to CIPR (Table 1B), which probably explains the comparably high amounts of Ox-Al and Ox-Fe in TSP treatments.

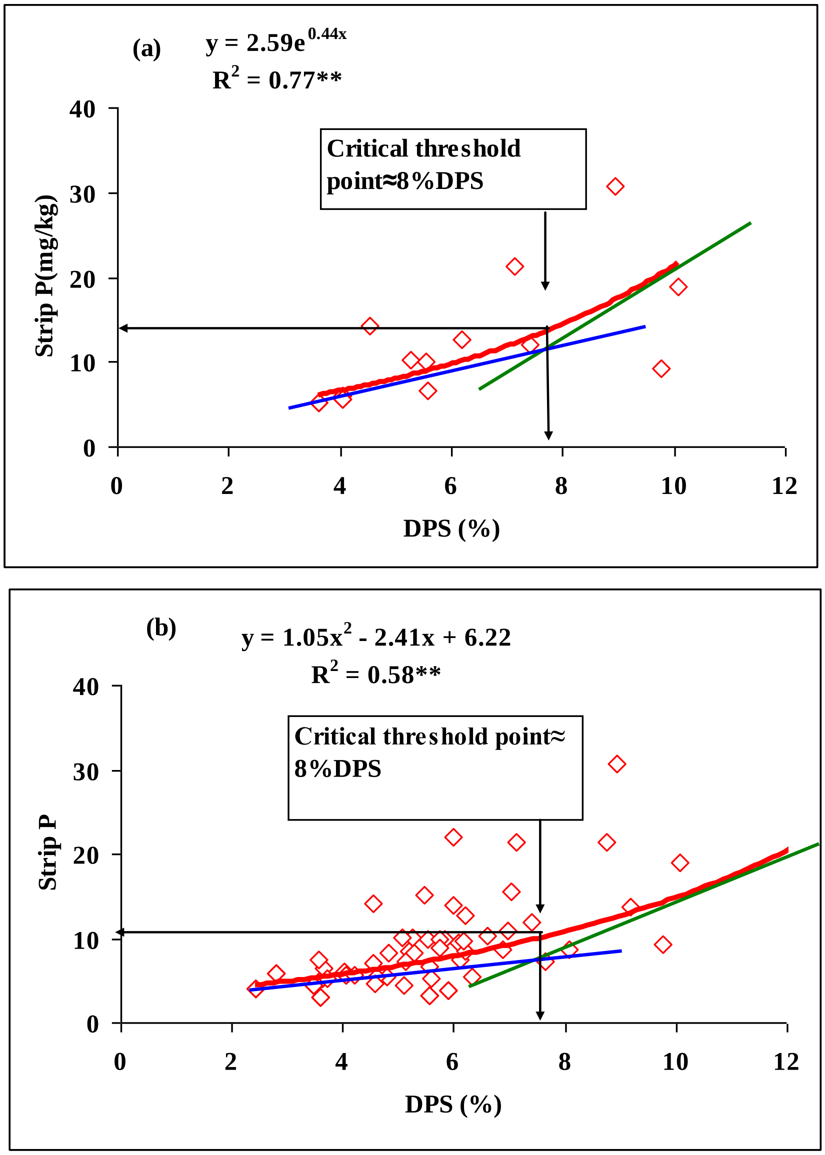

**FIGURE 6.** Relationship between (a) initial strip P vs. initial DPS (the first determined 2 months after treatment application); (b) average strip P (determined in five samplings) vs. average DPS determined for the five samplings. Note: the blue and green lines in a and b are the tangents for the lower and upper slopes, respectively. The point where the two intersect is considered the change point of the slope and the corresponding strip P is the critical P intensity.

On the other hand, Ox-Al and Ox-Fe decreased with the rate of P application due to formation of more stable complexes with P; hence, reducing the amounts of Ox-Al and Ox-Fe[24]. This was evident in the decrease in Ox-Al from 2673 to 2438 mg Al $kg^{-1}$, while Ox-Fe decreased from 3692 to 3660 mg Fe $kg^{-1}$ when the fertilizer rate was increased from 0 to 200 mg P $kg^{-1}$. At high P rates, PR dissolution decreased as shown by the lower rate of change in Ox-P extracted (0.79 mg $kg^{-1}$) as P application rates increased from 200 to 400 mg P $kg^{-1}$ compared to the higher rate of change in Ox-P extracted (0.88 mg $kg^{-1}$) as P application rates increased from 0 to 200 mg P $kg^{-1}$. Consequently, at the higher fertilizer rate, there was less P available for Ox-Al and Ox-Fe fixation, and therefore the levels of oxides increased in the soil at the 400 mg P $kg^{-1}$ rate. The high levels of Ox-Al and Ox-Fe in sole manure treatment were attributed to manure mineralization releasing some amorphous Al and Fe in high levels due to the high rate of manure applied (20 mg $ha^{-1}$ equivalent) compared to the rate of fertilizers applied (highest = 400 mg $kg^{-1}$). A combination of manure and the fertilizers led to an average increase of Ox-Al and Ox-Fe.

The magnitudes of Ox-P extracted were in the order: TSP > GPR > CIPR, consistent with the solubility of the fertilizers (Table 1B). This implies that as the P dissolved, it was susceptible to fixation by the amorphous Fe and Al in the soil[25]. Linear and quadratic relationships in TSP and PRs, respectively, were exhibited by plots of Ox-P against P rate. While TSP continued to dissolve linearly without any limitation, dissolution of PRs to release Ox-P increased to a maximum around 200 mg P $kg^{-1}$ and did not increase significantly afterwards. According to Chien and Black[26], the solution P of a PR is fixed at a maximum value for each PR regardless of how much PR is added to the soil. Consequently, the 200 mg P $kg^{-1}$ level probably corresponded to the specific solubility constant for CIPR and GPR used in this study.

The interactions of the P sources × manure × P rate showed varying amounts of Ox-Al and Ox-Fe with different P sources at different rates, which was attributed to the contents of these elements in the P sources, rate of dissolution, and the complex formation between P released/applied and the soil constituents. The high level of Al+Fe-Ox (≈7530 mg $kg^{-1}$) in the manure plots compared to lower levels in the control (≈5200 mg $kg^{-1}$) showed manure decomposed to release some amorphous Al+Fe-Ox concomitantly with P. The combination of manure and P fertilizers at 100 mg $kg^{-1}$ resulted in lower amounts of Al+Fe-Ox, particularly in TSP and CIPR (5950 mg $kg^{-1}$) due to complexion of the two oxides with P, but as the P rate increased to 200 mg P $ha^{-1}$, the released sesquioxides from the PR dissolution added to the levels, thus raising them, and this continued up to the 400 mg P $kg^{-1}$ rate. However, in GPR treatments, the continued decline of Al+Fe-Ox linearly with increasing fertilizer rates to a minimum (4640 mg $kg^{-1}$) at 200 mg P $kg^{-1}$ and the fact that it remained constant up to 400 mg P $kg^{-1}$ could be attributed to fixation of Al+Fe-Ox by the P released from PR dissolution and amounts remained low because of the low sesquioxide content in GPR (Fig. 1a). However, due to decline in GPR dissolution above the 200 mg P $kg^{-1}$ rate, Al+Fe-Ox did not decline or increase substantially above that level due to the lower P release rate (Fig. 1b).

The fact that the highest DPS was exhibited by the TSP/manure combination, while CIPR had the lowest, DPS-Ox could be explained by the high Ox-P and reduced concentrations of Al+Fe Ox. For example, the TSP/manure combination gave the highest Ox-P and at the same time had relatively lower Ox-Al and Ox-Fe than sole TSP and hence the difference in DPS. On the other hand, GPR with and without manure both gave higher DPS than sole TSP (Fig. 1c), which can be attributed mainly to the lower Ox-Al and Ox-Fe concentrations in GPR relative to TSP. Finally, CIPR had the lowest DPS, which was attributed to the fact that it had the lowest Ox-P and substantially high Ox-Al and Ox-Fe. However, in all the P sources, manure addition enhanced DPS, probably through the additional P from manure mineralization.

### Oxalate-Extractable Fe, Al, P, and DPS in the Field-Sampled Soils

Contrary to the closed incubation system, soils from the field indicated that P sources and manure did not influence Ox-Al and Ox-Fe significantly, probably because the rates of fertilizers used in the latter were lower (100–300 kg P $ha^{-1}$) compared to the rates used in the incubation trial (100–400 mg P $kg^{-1}$, equivalent to about 200–800 kg P $ha^{-1}$). Thus, on average, the amounts of Al+Fe-Ox from the different sources did not differ significantly. In addition, the open system seems to have led to increased PR dissolution as evidently shown by the equal amounts of P from CIPR and GPR (80 mg P $kg^{-1}$) in Table 2B. Dissolution increase in the open system could be attributed to removal of dissolution products, such as Ca, by leaching/plant removal and P removal by plants. On the other hand, the high solubility of TSP

may have led to complex formation between the Al+Fe-Ox and P, thus keeping the levels slightly higher than the PRs, but not significantly.

However, examining the different P sources for the first 6 months at the low fertilizer rate (100 kg $ha^{-1}$) revealed a declining trend followed by an increase that could be explained by an initial reaction between Fe and Al, with P leading to a decline of the three elements. Concurrently, the plants were also competing with the sesquioxides for P and as P decreased in the soil solution, some of the fixed P probably underwent dissolution, releasing P for plant uptake and concomitantly releasing the sesquioxides, causing their levels to rise again (Fig. 4b). At the highest fertilizer P application rate (300 kg P $ha^{-1}$), Al+Fe-Ox levels from the TSP were higher than in the PRs, probably due to lower dissolution of the PRs at the high fertilizer rate. Thus, the PRs released lower levels of Al+Fe-Ox and also lower levels of Ox-P (Fig. 4d), which could otherwise complex with the sesquioxides further lowering their levels. However, similar trends to those in the lower P rate were observed.

### DPS in both Incubation and Field Trial

The significant influence of fertilizer P sources on DPS in both the incubation and field experiments can be attributed to the different amounts of P, Ox-Al, and Ox-Fe released by the dissolution of the different fertilizers. The highest DPS in the incubation experiment was from GPR due to the relatively lower Al+Fe-Ox levels than the other sources (Fig. 1a). This is evidently shown by the fact that although TSP gave more than double Ox-P compared to GPR at 400 kg P $ha^{-1}$, the latter still exhibited much higher DPS compared to TSP (Fig. 1c). Accordingly, the lowest DPS was shown by CIPR treatments due to the fact that it had the lowest Ox-P and as high Al+Fe-Ox levels as TSP. The high DPS in incubated soils compared to the field-sampled soils could be ascribed to the higher levels of fertilizers (about 0–800 kg P $ha^{-1}$) applied in the incubated soils, which gave higher Ox-P levels of about 65–800 mg P $kg^{-1}$(Fig. 1b), which was almost threefold compared to the field Ox-P levels of about 60–280 mg P $kg^{-1}$ (Fig. 4c and d). On the other hand, the Al+Fe-Ox levels in incubated soils were 4500–8300 mg $kg^{-1}$ (Fig. 1a), just about double those in the field soils (2300–3800 mg $kg^{-1}$); hence, explaining further why DPS was lower in the field than incubated soils. The Ox-P was relatively lower in the field probably due to plant P uptake, which was absent in the laboratory study. The quadratic relationship between DPS and fertilizer rate in the incubated soils is attributed to the decreased dissolution of the PRs as shown by the lower amounts of Ox-P above 200 mg $kg^{-1}$ (Fig. 1b). The lower PR solubility at high rates is associated with attainment of a maximum solubility constant value characteristic to specific PRs[26]. At 300 kg P $ha^{-1}$ (field soils), the DPS for the two PRs were much lower than TSP due to the lower PR dissolution as discussed above. Thus, DPS integrated the effects of the sesquioxides, which influences the PSC of the soils and P into a single factor influencing P availability/release.

### P Sequentially Extracted Strip P from the Incubated Soil

GPR-treated soils released the highest cumulative total strip P, but it was not significantly different from TSP ascribed to the high P fixation of TSP, evident from the fact that it exhibited the highest Ox-P. Alternatively, sole CIPR had the lowest strip P released due to its low solubility. This observation is in agreement with the findings from the current work and previous experimental results that showed that direct application of reactive PR, such as GPR, produced comparable yields to those of TSP, while the nonreactive PRs, such as CIPR, were comparatively poorer[27].

The TSP/manure combination released the highest strip P in the first extraction, but declined rapidly, so that the GPR/manure combination had the highest after the initial extraction through all to the ninth strip. This implied that higher P was released by GPR than TSP and CIPR. Enhancement of strip P released by manure addition was more pronounced on GPR and CIPR, probably through improved PR dissolution. The different equations describing P release from TSP and PRs indicated a more rapid P

release in TSP than GPR. However, manure addition improved P release significantly for both PRs. Exponential-type equations described P release from both PRs and TSP. Similar findings of P release by strip P being described by exponential-type equations have been reported[28]. However, P release from PR-treated soils using strip P release had not been studied before.

### Relationship between DPS and Strip P Release

The good relationship and establishment of critical threshold points for P release when initial strip P was plotted against DPS and for the plot of total strip P against DPS for the incubated soils reflected the possibility of predicting potential P release from fertilized soils using either the single strip P or the total strip P. The strong relationship between initial P and total P is evidence that the two are suitable in predicting P release and, consequently, there would be no need for repeated or sequential extraction, but a single extraction would suffice. This is further supported by the fact that both the initial and total P gave almost similar threshold levels (21–22% DPS) and similar P release levels of about 13 mg P $kg^{-1}$. Similar to the incubated soil results, the field results exhibited similar relationships between strip P and DPS. They were also described by exponential functions showing distinct change points in the two parts of the curve, signifying DPS threshold points. However, the lower threshold DPS values of about 8% established in the field compared to 21–22% from the incubation studies would partly be attributed to the rhizosphere effects in the field experiment, where P released may have been increased at lower DPS levels as a result of the action of root exudates and probably not extracted by the strip P[29]. The authors reported that root exudates increased the solubility of P in soils. However, the rhizosphere theory may not fully explain the disparity in threshold levels between the incubated and field-sampled soils. The P intensity corresponding to 8% DPS in the field studies was 11–14 mg P $kg^{-1}$, which was close to the 13 mg P $kg^{-1}$ established in the laboratory, implying that when the average strip P from the sampled soil is around 11–14 mg P $kg^{-1}$, P release should be monitored.

Comparison of our threshold DPS levels established to other studies must be cautiously done because the threshold levels have been shown to vary with the method of DPS and P release determination[30]. The strip P used in our study to determine P release has not been used in any other DPS threshold level study. The majority of the researchers used water-extractable P (WEP), while others used calcium chloride–extractable P ($CaCl_2$-P) to determine P release and ended up with different threshold DPS levels[30]. They established DPS levels ranging from 3 to 44% for WEP and 11 to 51% for $CaCl_2$-P in the soils they tested. Other soil P threshold levels reported varied from 10%[8] to 26–38%[31] in different soils and varying methods. The amounts of strip P extracted from the soils could probably be slightly higher than WEP or $CaCl_2$ if used in the test soils of our experiment. This is because water would be less efficient in extracting P from the water-insoluble PRs, while $CaCl_2$ would also be less efficient in extracting P from the PRs due to the common ion effect. However, the threshold DPS levels we established falls within the reported range. These results confirm the findings of other authors indicating that the fraction of surface coverage with P is the dominant soil parameter actively controlling the mechanism of P desorption[32]. The critical P release levels we established are just slightly above the strip P agronomic level (12 mg P $kg^{-1}$) required for optimal dry matter production[23].

## CONCLUSIONS

The DPS exhibited significantly high correlation with amount of initial and total P released from the strips, which confirms the findings of other authors indicating that the fraction of surface coverage is the predominant soil parameter actively controlling the mechanism of P desorption[27,31]. The results of this study also show that the sequential soil P extraction procedure using the strip P can be used to characterize the kinetics and potential ability of a soil to release P to runoff or subsurface drainage. In addition, a single-strip extraction of P is adequate in order to establish the critical DPS of the soil. The threshold DPS level of 21–22% established from incubated soils compared to the threshold level of 8% established in the field-sampled soils is evidence of the influence of environmental factors on the threshold DPS and therefore the need of establishing recommendations on field trials other than incubation studies. The fact that the DPS threshold corresponded to strip P release of 11–14 mg P $kg^{-1}$ implies that whenever strip P is determined from a field and it is within this range, there is need to determine the DPS in order to assess the P release capability. It is advantageous to use strip P to determine P release because the same test method was recommended for use in conventional and nonconventional P sources, such as PRs[32]. If using this test for fertility monitoring, then it means that when the strip P test levels are above 14 mg P $kg^{-1}$, it will be an alert to assess P release to the surface waters. Consequently, further research is recommended in different agroecological zones in order to establish some guidelines for practical applications of DPS to predict P release. In addition, it may be important to identify quick and more suitable routine methods for DPS determination, other than oxalate-extractable P, Fe, and Al.

### ACKNOWLEDGMENTS

We are grateful to the Institute of Tropical Agriculture for offering a postdoctoral fellowship and to the Third World Organization of Women in Sciences (TWOWS) for the Ph.D. fellowship offered to the first author, Esther W. Gikonyo. Finally, thanks to the staff of the Department of Land Management, Faculty of Agriculture, Universiti Putra Malaysia, where the research was conducted.
